# Editorial: Exploring the role of inflammation in depression

**DOI:** 10.3389/fphar.2026.1911946

**Published:** 2026-07-15

**Authors:** Savina Apolloni, Amanda Gollo Bertollo, Ricieri Mocelin, Zuleide Maria Ignácio

**Affiliations:** 1 Department of Biology, University of Rome Tor Vergata, Rome, Italy; 2 Graduate Program in Neurosciences, Federal University of Santa Catarina, Florianópolis, Brazil; 3 Translational Neuropsychobiology Laboratory, Graduate Program in Biomedical Sciences, Federal University of Fronteira Sul, Passo Fundo, Brazil; 4 Laboratory of Physiology, Pharmacology, and Psychopathology, Graduate Program in Biomedical Sciences, Federal University of Fronteira Sul (UFFS), Chapecó, Santa Catarina, Brazil

**Keywords:** gut-immune-brain axis, inflammation, major depressive disorder, neuroinflammation, treatment-resistant depression

## Introduction

The inflammation in major depressive disorder (MDD) permeates several states of the disease and is involved in treatment-resistant depression (TRD), among other conditions (Beurel et al., 2020; Chamberlain et al., 2019). In chronic systemic inflammation, altered biological processes can impair the blood-brain barrier (BBB). Chronic dysregulation of the immune system, with increased release of inflammatory cytokines, affects the BBB and the central nervous system (CNS), altering several neurophysiological mechanisms that can culminate in MDD and other psychiatric disorders (Beurel et al., 2020; Mancuso et al., 2023). Among the altered neurophysiological mechanisms, it is possible to highlight oxidative balance, dysregulation of neurotransmitter release and function, neuronal signaling pathways, and metabolic pathways, such as the kynurenine pathway, among others, which impair the structure and function of brain regions involved in mood regulation (Mancuso et al., 2023; Bertollo et al., 2024). Research has been highlighting that these altered mechanisms from inflammation can lead to MDD or aggravate depressive conditions, culminating in TRD (Beurel et al., 2020).

**FIGURE 1:**
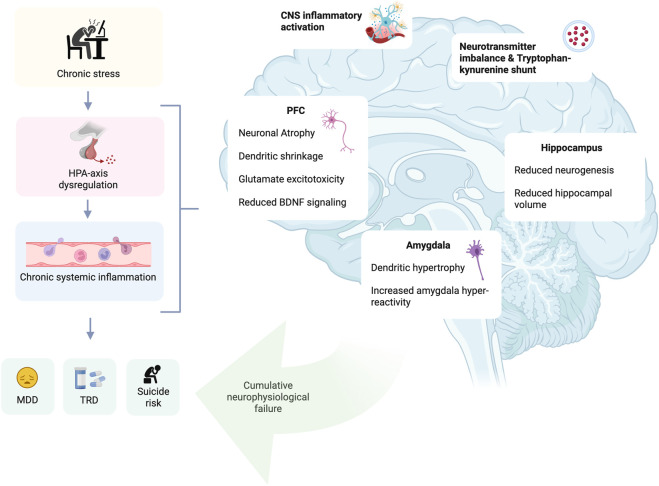
Schematic representation of the pathways linking chronic stress to depressive disorders via immune and neurophysiological dysregulation. Chronic stress triggers HPA-axis dysregulation, which subsequently leads to chronic systemic inflammation. This peripheral inflammation promotes central nervous system (CNS) inflammatory activation and drives neurotransmitter imbalances, notably through the tryptophan-kynurenine shunt. These alterations result in cumulative neurophysiological failures across specific brain regions, including neuronal atrophy, dendritic shrinkage, glutamate excitotoxicity, and reduced BDNF signaling in the prefrontal cortex (PFC); reduced neurogenesis and hippocampal volume loss in the hippocampus; and dendritic hypertrophy alongside increased hyper-reactivity in the amygdala. Ultimately, these structural and functional impairments contribute to the development of major depressive disorder (MDD), treatment-resistant depression (TRD), and an increased suicide risk.

This special Research Topic includes studies that combine original research and literature reviews, as well as a clinical trial, highlighting several crucial biological mechanisms and psychological factors related to the inflammatory process in depression (Figure 1).

Several contributions focus on the molecular and cellular mechanisms linking inflammatory processes to depressive symptomatology. Emerging evidence highlights oxidative stress, inflammasome activation, mitochondrial dysfunction, and alterations in lipid metabolism as key biological pathways that promote neuroinflammation and disrupt neural circuits involved in mood regulation. Together, these studies support the concept that depression arises from complex interactions among metabolic, immune, and neurobiological systems. Shi et al. combined proteomic and transcriptomic approaches to investigate anxious depression and identified the ROS/TXNIP/NLRP3 signaling pathway as a critical link between mitochondrial oxidative stress and neuroinflammation. Their findings highlight inflammasome activation as a promising target for future interventions. Similarly, Li et al. demonstrated that TLR4-mediated activation of the kynurenine pathway contributes to post-traumatic depression in a murine traumatic brain injury model, providing mechanistic evidence linking immune signaling, neuroinflammation, and depressive-like behavior. The contribution of metabolic dysregulation to inflammatory pathways was further explored by Liu et al. who examined the comorbidity risk characteristics of rheumatoid arthritis in the context of depression-associated lipid metabolism, supporting the existence of shared inflammatory and metabolic mechanisms across psychiatric and immune disorders. Along the same lines, Ge et al. reviewed the role of lipid metabolic dysregulation in MDD, emphasizing how alterations in lipid homeostasis may promote neuroinflammatory responses and contribute to disease pathophysiology. Complementing this perspective, Garcia-Juarez et al. discussed the roles of ceramides and neuroinflammation as immunometabolic drivers and biomarkers of MDD, TRD, and suicidal vulnerability, highlighting the growing relevance of lipid signaling pathways in neuropsychiatric research.

Another important theme in this Research Topic concerns TRD. Persistent neuroinflammatory states may contribute to reduced responsiveness to conventional antidepressant therapies. Inflammatory mediators, glial dysfunction, and immune-related metabolic pathways are emerging as promising therapeutic targets. In this regard, Miyata et al. provided a comprehensive mini-review of neuroinflammation in major depressive disorder. They summarize current evidence supporting neuroimmune dysfunction as a central mechanism underlying treatment resistance and recommend targeting neuroinflammatory pathways and neuroimmune dysfunction in future therapeutic strategies.

The Research Topic also expands the understanding of inflammation-related risk factors across the lifespan. Zhang et al. investigated the associations among childhood trauma, clinical characteristics, and inflammatory cytokines in adolescents with first-episode and recurrent MDD. Their findings support the hypothesis that trauma-related immune activation may be important in the initial onset and may increase vulnerability to depression. Furthermore, studies examining suicidal behavior identify associations between inflammatory biomarkers and clinical outcomes. This suggests that immune alterations may contribute to the severity and complexity of depressive disorders. Şimşek et al. conducted a retrospective cohort study examining the relationship between suicidal behavior and hematological inflammatory parameters. Their work adds to the growing evidence that peripheral inflammatory markers may represent accessible and low-cost biomarkers in the assessment of suicide risk.

Beyond mechanistic investigations, several articles explore innovative therapeutic perspectives. Simões et al. reviewed the gut-immune-brain axis in inflammatory bowel disease and depression, recommending that future therapies focus on targeting neuroimmune communication pathways and addressing peripheral inflammation to improve depressive symptomatology. Pilatti et al. examined the interplay between the hypothalamic-pituitary-adrenal axis and inflammation, suggesting that compounds from *Cannabis sativa* warrant further investigation as potential anti-inflammatory and neuroprotective agents. Finally, Moloney et al. presented a randomized, double-blind, placebo-controlled clinical trial evaluating low-dose naltrexone as an adjunctive treatment for major depressive disorder. Although the intervention did not show significant benefits over placebo, the study provides valuable clinical evidence for ongoing investigations of immunomodulatory strategies in depression and highlights the importance of developing more targeted approaches to neuroinflammation-based treatments.

## Discussion and conclusion

The present Research Topic of studies consolidates the view that MDD and TRD arise from complex interactions among metabolic, immune, and neurobiological systems. Chronic immune dysregulation and excessive release of inflammatory cytokines directly affect the BBB and CNS. This process compromises brain structures and functions involved in mood regulation by disrupting multiple neurophysiological mechanisms.

The studies gathered in this Research Topic reinforce that chronic inflammation and neuroimmune dysfunction play a central role in the pathophysiology of depression, actively contributing from symptom onset to the development of treatment resistance.

Although the randomized controlled trial evaluating adjunctive low-dose naltrexone did not demonstrate statistically significant superiority over placebo, its findings provide valuable clinical insights for advancing immunomodulatory strategies. The outcome of this investigation underscores an urgent need in contemporary biological psychiatry: the development of more targeted and personalized therapeutic approaches based on the specific neuroinflammatory profiles of individual patients.

Ultimately, the future management of major depressive disorder will depend on our ability to precisely modulate neuroimmune communication pathways and mitigate peripheral inflammation, thereby opening more effective avenues to alleviate depressive symptoms and restore patients’ quality of life.
